# Reconstruction of Velocity Curve in Long Stroke and High Dynamic Range Laser Interferometry

**DOI:** 10.3390/s21227520

**Published:** 2021-11-12

**Authors:** Jinbao Feng, Jinhui Wu, Yu Si, Yubin Gao, Ji Liu, Gao Wang

**Affiliations:** 1 School of Information and Communication Engineering, North University of China, Taiyuan 030051, China; b1905043@st.nuc.edu.cn (J.F.); s1905038@st.nuc.edu.cn (Y.S.); liuji6@nuc.edu.cn (J.L.); wanggao@nuc.edu.cn (G.W.); 2 School of Information Technology Application and Innovation, Yuncheng Vocational and Technical University, Yuncheng 044000, China; 3 School of Instrument and Electronics, North University of China, Taiyuan 030051, China; wujinhui@nuc.edu.cn; 4 School of Science, North University of China, Taiyuan 030051, China

**Keywords:** photonic Doppler velocimetry, ridge extracting correction algorithm, large displacement speed measurement

## Abstract

To study the law that governs the complex movements of the mechanism in the process of automatic weapon operation, the velocity tracking test technology of photon Doppler velocimetry is introduced to accurately measure velocity, displacement and acceleration, on the condition that there are long displacement and rapid velocity change. In the traditional way, out of interference signal time-frequency (TF) transformation draws TF distribution, and then by modulus maxima frequency extraction, comes to the law of velocity change. Due to the influence resulting from the change of fundamental signal as well as that of light intensity signal in the test, based on the TF distribution obtained by TF transformation, the traditional modulus maxima frequency extraction can extract frequency signals, but they show abnormal sudden changes at some moments, making the velocity discontinuous, unsmooth and unreal, which brings obvious errors to the subsequent calculation of acceleration and accurate displacement. Addressing the above-mentioned problems, this paper proposes a ridge extracting correction algorithm based on modulus maxima frequency extraction; this method, based on a large number of experiments where rodless cylinders are used to simulate the motion of a gun automatic mechanism, conducts a detailed calculation and analysis of the experimental results. A comparison of the two algorithms’ processing results, in terms of the speed, displacement and acceleration, suggests that the ridge extracting correction algorithm successfully corrects the frequency selection error, which draws a more continuous and, therefore, effective curve of the velocity change, and by so doing, the error of the displacement test (within 1.36 m displacement) is reduced from more than 3.6% to less than 0.58%, and the uncertainty dropped 97.07%. All these show that the accurate measurement of velocity, displacement and acceleration, with sudden and rapid velocity changes considered, is realized successfully.

## 1. Introduction

Because of the explosive influence and mechanical coupling effects, there are complex laws of impact, friction and vibration in the velocity tracking test, such as the motion law of the gun automatic mechanism and the velocity change of the gun back seat. Therefore, it has the characteristics of rapid changes in velocity, short periods and long displacements. At present, target velocity tracking test methods include high-speed photography, microwave radar and laser Doppler velocity measurement. Among them, microwave radar has poor directivity and needs a relatively large moving surface to receive the reflected signal. High-speed photography often fails to capture objects and suffers from image blurring and image oversaturation. Photonic Doppler velocimetry (PDV) only needs a single light pointed on the object surface to be measured and obtains the speed according to the return light Doppler frequency shift interference fringes. It has the advantages of high precision, high reliability, and reusability. Compared with the traditional laser speed measurement system, the PDV [1,2,3,4,5] speed measurement system has the advantages of strong anti-disturbance ability, large speed measurement range, good robustness of measurement results, etc., and is more suitable for vibration, impact and other measurement occasions with low signal-to-noise ratio and poor signal quality, such as high temperature, high speed and high pressure [6]. Since the year 2004 when the conventional PDV system was proposed by O.T. Stand using a three-port circulator, PDV systems have evolved rapidly in four aspects: larger velocity range, higher time resolution, higher sensitivity, and multi-point measurements. For example, in 2019, J.G. Mance and B.M. La Lone et al. [7] proposed time-stretched PDV, which transfers spectral information to the time domain and there it, by means of fast photodetectors and digitizers, records data quickly. In 2020, Yohan Barbarin [4] built a 16-channel multiplexed crosstalk-free multiplexed PDV (MPDV), which reduces the total number of fiber components through WDM and also overcomes the crosstalk issues caused by reducing the mesh size. In 2021, Chu [8] constructed a time-lens to extend the dynamic range of the PDV system, called the time-lens PDV system, which successfully reduces the frequency range from 120 GHz to 12 GHz, extending the speed measurement range by 10 times; and it is suitable for ultra-high speed. In 2021, A.V. Pavlenko et al. [9] constructed a hybrid interferometric system for measuring the surface velocity of targets in shock wave experiments. The system integrates an all-fiber velocity interferometry system for any reflector with a PDV system. This hybrid interferometric scheme makes it possible to measure the surface velocity at the same point, simultaneously with two independent devices. Unlike the issues faced by the above-mentioned methods, the problem we face is how to select the correct signal by avoiding harmonic interference in the STFT results of a single point test; obviously for this the above-mentioned methods do not work.

Signal demodulation is an important part of the PDV system. Its purpose is to demodulate the velocity information of the measured object from the Doppler signal with considerable noise. At present, there are four demodulation methods [10,11,12,13,14,15,16,17] for the signal of a PDV system: the fringe method, short-time Fourier transform (STFT) method, wavelet transform method [18,19], and phase demodulation method. In 2012, Song. and Wu. et al. [20] discussed the performance of the STFT and the continuous wavelet transform (CWT) in processing fast-changing low-speed fringe signals measured by PDV through experiments. In the test based on the Kolsky-bar, both methods show effectiveness of processing the longitudinal velocity signal of the bar. However, the CWT cannot correctly analyze the radial velocity of the impact sample, which indicates that the CWT is sensitive to background noise, and the STFT has strong robustness in extracting low Signal to Noise Ratio (SNR) waveforms. In 2019, Dai. et al. [21] discussed the principles, characteristics and applicable conditions of the four demodulation methods: the fringe method, phase demodulation method, STFT method and wavelet transform method based on the principle of a PDV system. The principle of the fringe method is simple, and the demodulation process is reliable, but the calculation steps are cumbersome, and the demodulation error is serious when the fringe is sparse. The wavelet transform has an adjustable window width and high TF resolution, but its disadvantage is that the wavelet basis function cannot be changed after it is determined, and the choice of the wavelet basis function depends largely on experiments and experiences; thus, it is uncertain. The phase demodulation method has high resolution, but it requires high signal quality and easily introduces quadrature error. The STFT algorithm has the lowest complexity, the fastest operation speed and low requirement for signal quality. Therefore, the STFT algorithm should be used to demodulate the signal in the speed tracking test environment of the gun automatic mechanism, which is characterized by long motion displacement and velocity jump, then the TF curve could be extracted from the demodulated TF distribution.

Owing to the effects of the fundamental signal, light intensity signal and noise, there are always frequency jump phenomena that are not caused by acceleration when using the STFT in actual tests, which causes obvious errors in the post calculation of acceleration and displacement. Among all the postprocessing methods of STFT results, the synchrosqueezing transform (SST) and the synchroextracting transform (SET) are commonly used. The SST is to squeeze all TF coefficients into the instantaneous frequency (IF) trajectory. Differ from the squeezing manner of SST, the main idea of SET is to only retain the TF information of STFT results most related to time-varying features of the signal and to remove most smeared TF energy, such that the energy concentration of the novel TF re-presentation can be enhanced greatly. Both algorithms emphasize the energy enhancement but not finding and correcting the wrong information [22]. At present, the methods that can correct the error points of the frequency signal extracted by the modulus maxima frequency extraction (MMFE) method, are the curve fitting method, the interpolation method, the SAVER algorithm [23] and the MeanShift algorithm [24]. Both the curve fitting method and the interpolation method correct the velocity curve mutation term (jump point) according to the algorithm after extracting the velocity curve, and neither of them can extract the real velocity of the jump point. SAVER relies on importing and cropping data to the time and velocity range of interest, and users can choose to manually crop spectrograms or set time and velocity limits for more accurate, objective and fast PDV data analysis. SAVER was generally unaffected by artifacts in the input spectrograms, but scatter in the extracted results was seen to increase as the input signal quality turns worse. This method requires manual selection and is computationally intensive, while the results are influenced by the input signal, so it is unsuitable for the correction of speed values in large-stroke velocity measurements. The MeanShift algorithm is based on the STFT of the original PDV signal, the intensity threshold filtering and binarization of the generated TF matrix to obtain the matrix, and then the connected domain is extracted from the data blocks in this matrix. According to the constraint that the change of the slope of the trajectory cannot be greater than a certain threshold, a search direction constraint chain table consisting of the center of gravity of the matrix connected domain is given. Finally, because the MeanShift iterative process has a feature that it always points to the direction with the largest increase in the probability density function, the feature is utilized to perform a trajectory search of the matrix for valid signals. A weight function associated with the change in the slope of the trajectory is introduced to guide the search along the direction given by the search direction constraint chain table. The algorithm is not suitable, because on the one hand it requires filtering and binarization of the intensity, and on the other, it is complex and computationally intensive, while the focus in the large stroke excitation velocity measurement is on the velocity from the beginning to the end of the whole velocity change process.

Therefore, this paper proposes a new method called ridge extracting correction algorithm (RECA), a ridge extracting correction algorithm based on STFT. This algorithm completes FFT within a window length range, taking the frequency of the current FFT as the center point, and uses a special weighting function to construct a frequency weighting value. Therefore, when the next FFT is performed within a window length range, each spectrum module is multiplied by the corresponding weighting value, and the frequency corresponding to the maximum value of the new spectrum module is the frequency of this FFT. This method solves the above mutation problem, making the speed curve of the long stroke continuous velocity measurement more continuous, smoother and more accurate, and provides the basis for the subsequent extraction of parameters (acceleration and displacement).

## 2. System Theory Analysis

### 2.1. Optical Path Analysis and Design

Because the PDV is based on the Michelson interferometer model, there is a certain requirement for the PDV system to couple the power of the signal light but no requirement for imaging.

According to the light intensity response principle of the photodetector, the interference signal generated by the photodetector can be expressed as:

(1) $$P\left(t\right)=\frac{d}{2}\left(A_{0}^{2}+A_{1}^{2}\right)+dA_{0}A_{1}\cos[\left(\omega_{0}-\omega_{1}\right)t+\left(\varphi_{0}-\varphi_{1}\right)]$$

where *d* is the response coefficient of the system detector, *A*_0_ and *A*_1_ are the amplitudes of the reference light and the signal light respectively, and *ω*_0_, *φ*_0_, *ω*_1_, and *φ*_1_ are the initial angular velocity, initial phase, signal angular velocity, and signal phase, respectively. Formula (1) shows that the expression (second term) contains effective information that is only related to *d*, *A*_0_ and *A*_1_. Because the PDV optical path structure ensures that the reference light has a stable signal intensity, even if the quality of the signal light is attenuated due to the motion attitude of the object, optical parameters and coupling efficiency, the signal-to-noise ratio of the interference signal can be ensured by increasing the signal intensity of the reference light. The relationship between the frequency of the interference signal and the moving speed of the object is as follows:

(2) $$f=\left|\frac{\omega_{0}-\omega_{1}}{2\pi }\right|=\frac{2u}{\lambda_{0}}$$

where *u* is the velocity of the object moving in the opposite direction of the laser propagation direction and *λ*_0_ is the laser wavelength. The detector with AC coupling output can filter out the first DC component in Formula (1) and reduce the signal saturation.

In the practical application of PDV, because of the small numerical aperture of single-mode fiber and the divergence angle of the laser beam from fiber aspherical lens coupling, in the dynamic test, the effective coupling space of the coaxial optical system is severely limited and is only limited within the paraxial region. To improve the coupling efficiency, a micro prism infrared reflective film with 0.57° divergence angles is pasted on the surface of the measured object. The diameter of the optical fiber aspherical lens was 3 mm, the output laser was 60 mW, the spot size was 8.5 mm, as measured by an infrared photosensitive detection card at a distance of 1.5 m from the lens surface, and the optical power was 48 mW. Place a reflecting film on the optical path and define the optical signal output from the 3rd port of the circulator as static signal. When the distance between the lens and the reflecting film is 150 cm, the optical power of the 3rd port static signal is 20–30 μW. The static signal optical power can reach 260 μW at 30 cm. After Erbium Doped Fiber Amplifier (EDFA) amplification and filtering, the response condition of the detector is satisfied. The noise of the laser system and the detector is 8 mV. The SNR of the interference signal at 150 cm is measured by means of micro disturbance, and the result is 16 dB ≤ SNR ≤ 23 dB, which meets the requirements of signal demodulation.

**Figure 1:**
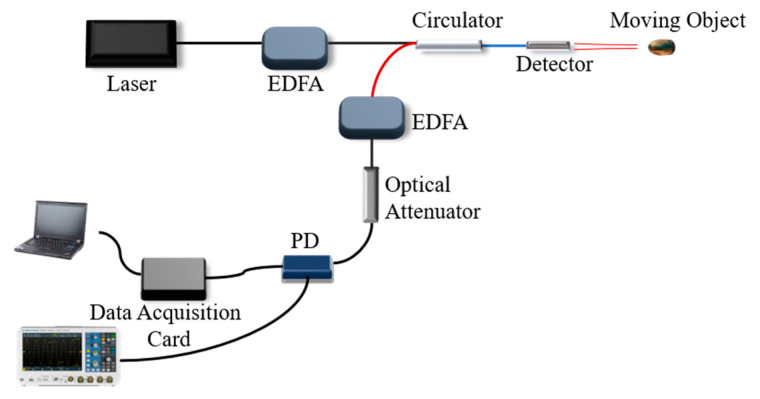
Schematic diagram of the system light path structure.

The system is designed as an all-fiber optical structure. In the method, light reflected from the optic fiber end face interferes with the light returning from the tested objects, namely, the heterodyne method, as shown in Figure 1. The 1550 nm laser with 14 mW power of a 20 kHz narrow linewidth laser enters the circulator after being amplified by an EDFA. An aspheric lens with a working distance of 300 mm is used to emit the laser to the surface of the object to be measured, and the output laser power of the probe is 60 mW by using a space optical power meter. At the same time, a part of the reflected light is generated at the connection between the circulator’s 2nd port and the aspheric mirror as the reference light [25,26], the frequency of which is still *f*_0_. The laser, with a shifted frequency, is generated by the reflection of the object surface, which is called the signal light, having a frequency change *f*_d_. The signal light is coupled to the circulator’s 2nd port again through the aspheric mirror and enters the circulator’s 3rd port together with the reference light. The interference signal light amplified by EDFA is filtered by the square law detection of the photodetector and AC coupling output. Finally, the interference fringe signal is recorded by an oscilloscope or by a data acquisition card, and the velocity signal can be obtained through PC after data processing.

In this system, the interferometer offers a common optical path for both signal light and reference light (as shown in Figure 1), which has stronger anti-disturbance ability than an interferometer with signal light and reference light passing through different optical paths.

According to the above system design scheme, the velocity can be obtained by the Doppler frequency shift formula:

(3) $$u\left(t\right)=\frac{\lambda_{0}}{2}\left(f_{d}-f_{0}\right)=\frac{\lambda_{0}}{2}f_{m}\left(t\right)$$

where *u*(*t*) represents the function of velocity with time, *λ*_0_ represents the wavelength of the laser, *f*_0_ represents the laser frequency, *f_d_* represents the laser frequency with Doppler frequency shift, and *f_m_*(*t*) represents the function of fringe frequency changing with time.

### 2.2. Signal Processing

#### 2.2.1. Traditional Modulus Maxima Frequency Extraction Algorithm

The STFT method is adopted in the system. The STFT is an enhanced mathematical method derived from the discrete Fourier transform (DFT), which is used to study the instantaneous frequency and amplitude of local waves with time-varying characteristics [27]. The basic idea of the STFT is to assume that the nonstationary signal is a piecewise stationary signal, intercept the signal with a fixed width sliding window, perform an FFT for each section of the signal, and obtain the spectrum of each section of the signal. The frequency of each section of the signal is the frequency corresponding to the maximum of the spectrum modules. The type and width *N* of a window, which directly affects the frequency resolution *f_res_*, should be taken into consideration before doing signal segmentation analysis. The relation between *N* and *f_res_* can be described as:

(4) $$f_{res}=\frac{f_{s}}{N}$$

where *f_s_* represents the system sampling rate.

In the calculation, there is an *L_ov_* long overlap between the former and the latter window, which can improve the time resolution of TF signal, making the TF curve more continuous, and avoid occurring velocity jumping-points. Time resolution *t_res_* can be described as:

(5) $$t_{res}=\frac{N-L_{ov}}{f_{s}}$$

In addition, according to the characteristic that the energy distribution of laser interference signal is relatively concentrated, the Hamming window with smaller sidelobe component is used in this experiment to reduce the influence of spectrum leakage. The actual frequency *f* of the analyzed signal can be obtained by the modulus maxima frequency extraction (MMFE), the algorithm of which can be described as Equation (6):

(6) $$ridgeline\left(t\right)=\arg\underset{a\in R}{\max}\left|STFT\left(t,\omega \right)\right|$$

From Equation (6), the whole *ridgeline* can be extracted from *STFT* result, which could be substituted into Equation (7) to get a time-frequency curve:

(7) $$f\left(t\right)=\frac{ridgeline\left(t\right)\cdot f_{s}}{N}$$

where *f_s_* is system sampling rate, *N* represents the number of points involved in the Fourier transform. The velocity of the object is obtained as a function of time, *u*(*t*)*,* over its entire range of motion.

#### 2.2.2. RECA Based on STFT

**Figure 2:**
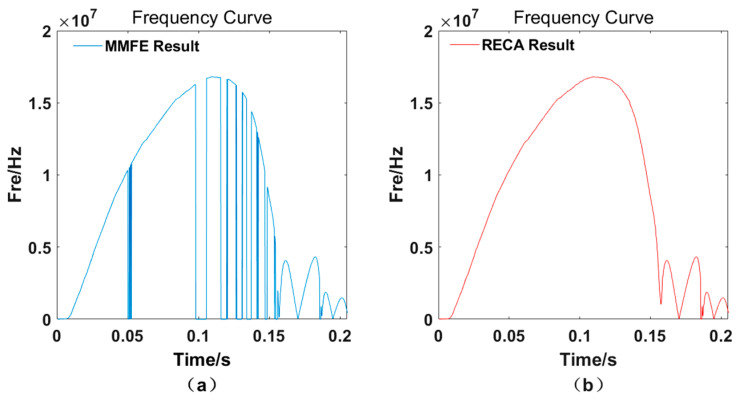
Comparison of the speed curve of the rodless cylinder throughout its motion. Speed curve processed by (**a**) MMFE method and by (**b**) RECA method.

Due to the influence of the fundamental wave signal and light intensity signal changes, after processing the interference signal data with the traditional MMFE, there are always frequency jump points on the curve, simply because of incorrect frequency selection, even though the acquired signal is relatively stable. Taking the whole movement process of the gun automatic mechanism simulated by a rodless cylinder as an example, the speed curve after the traditional MMFE is shown in Figure 2a.

The traditional methods to solve this problem include interpolation and curve fitting, but all of these algorithms add data points according to the trend of speed change after calculation, which cannot reflect the real frequency of speed at the time of the frequency jump. Therefore, the RECA is proposed to solve the problem of frequency jumps in the data processing, making the calculation results more real and accurate, as shown in Figure 2b.

**Figure 3:**
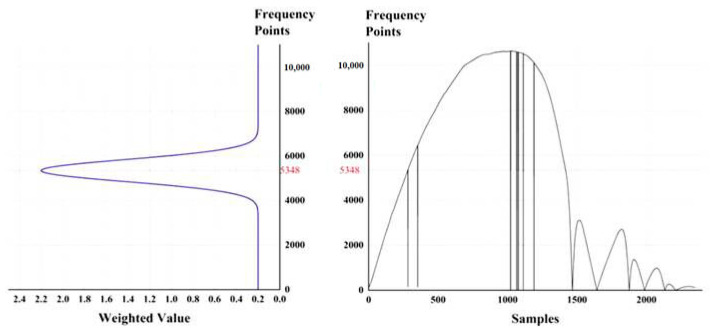
Distribution of weighted values of sample points.

The main idea of the algorithm is to construct a special weighted function *k*(*x*), that is, a constant function plus a Gaussian function. The form is as Follows (8):

(8) $$k\left(x\right)=q_{b}+q_{jd}e^{-{\left(x-d_{fc}\right)}^{2}/2q_{jk}^{2}}$$

where *q_b_* is the self-weight, that is, the value of the constant function; *q_jd_* is the weighted degree, that is, the peak value of the Gaussian function; *q_jk_* is the weighted width, which is the standard deviation of the Gaussian function, also known as the Gaussian RMS width, and *d_fc_* is the frequency point selected in the last Fourier transform, which is the intermediate frequency point of this transform, that is, the symmetric center point of the Gaussian function. As shown in Figure 3, in the legend, the value of the self-weight *q_b_* is 0.2, the value of the weighting degree *q_jd_* is 2, the value of the weighted width *q_jk_* is 500, and the value of the intermediate frequency point *d_f__c_* is 5348, forming the weighting coefficient of each frequency energy value of the next *STFT*. The corresponding weighted distribution values of this point are shown in the left part of Figure 3.

The selection of each parameter in the equation above is recommended as follows. The function of the self-weight *q_b_* is to retain part of the corresponding original values of the spectral module signal corresponding to each frequency after FFT, that is, to multiply the weight *q_b_*, to avoid the phenomenon that the actual signals are submerged by the value weighted by the Gaussian function due to a frequency jump that is too large. The value of *q_b_* is selected according to the strength of the effective signal and the interference signal in the experimental test, and the value is between 0.1 and 0.2 according to experience. The coefficient *q_jd_* is the weighting degree and the peak value of the Gaussian function. The weighting effect is obvious in a certain range with *d_fc_* as the center and *q_jk_* as the width. According to experience, the value is 10 times *q_b_*, that is, the value is 1 to 2; the coefficient *q_jk_* is the weighted width, which determines the relative distribution range of the Gaussian function, which is between 500 and 1000 based on experience. The three parameters above can be adjusted relative to the actual signal interference.

The specific calculation method is as follows:

Step 1: Set each parameter of the weighting function *q_b_*, *q_jd_*, *q_jk_* and *d_fc_*, where *d_fc_* is the correct frequency point extracted from the MMFE calculation after the last windowed FFT.

Step 2: Assuming that the number of points involved in FFT is *L* = 2n, {n|n > 0, n ∈ N}, because the spectrum generated by FFT is conjugate symmetric, a row vector such as *L*/2 elements can be generated by weighted function Equation (8):

$$A=\left[a_{0},a_{1},\cdots ,a_{L/2-1}\right]$$

In detail:

$$a_{i}=k\left(i\right)=q_{b}+q_{jd}\exp\left[-{\left(i-d_{fc}\right)}^{2}/2q_{jk}^{2}\right]$$

Thus, a column vector *A* composed of *L*/2 elements is generated.

Step 3: For a traditional FFT, define the first *L*/2 elements as a row vector *B*:

$$B=\left[b_{0},b_{1},\cdots ,b_{L/2-1}\right]$$

Step 4: Row vector *C* is deduced from the row vector *A* and row vector *B*:

$$\begin{array}{c} C=\left[c_{0},c_{1},\ldots ,c_{L/2-1}\right]\\ =\left[a_{0}\times b_{0},a_{1}\times b_{1},\ldots ,a_{L/2-1}\times b_{L/2-1}\right] \end{array}$$

Step 5: The sizes of each value of the row vector are compared so as to reach the maximum value. Setting the maximum value as *c_m_*, the final frequency of this algorithm is *f* = *m* × *f_s_*/*L*, where f_s_ is the system’s 200 MHz sampling rate and *L* is the number of points participating in the Fourier transformation.

Step 6: The next calculation of the FFT frequency tracking algorithm of the next period point (L) is started by returning to the first step. The *d_fc_* value of the next calculation is the m value of this time.

**Figure 4:**
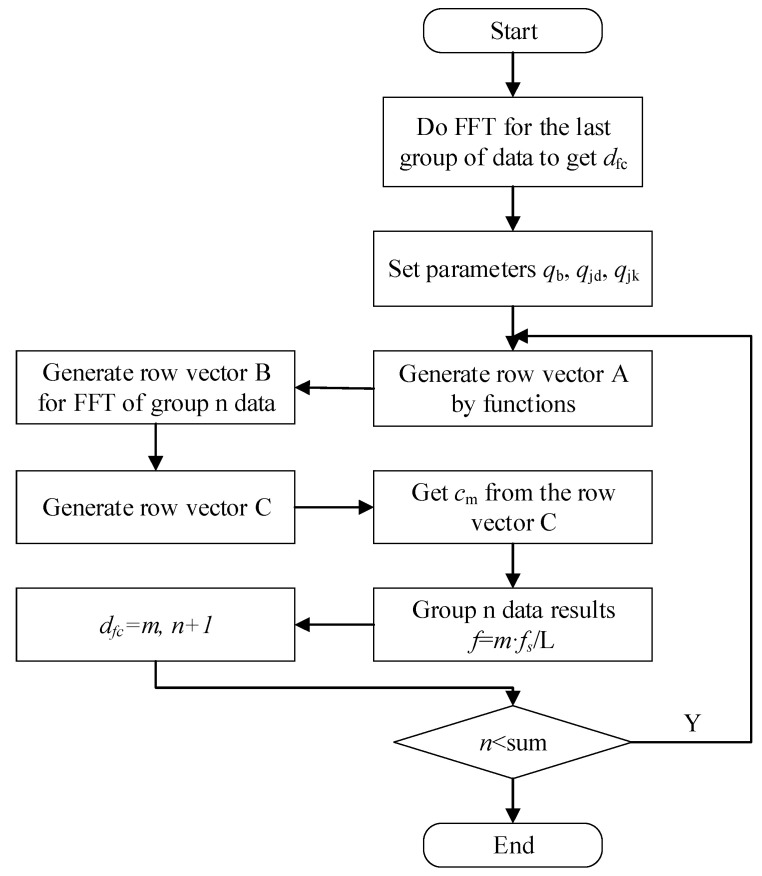
Flow chart of the RECA.

Algorithm flow chart is shown as Figure 4.

## 3. Experiment Construction and Result Analysis

### 3.1. Experimental Construction

In the experiment, the mechanical rodless cylinder is used as the velocity source to replace the automatic mechanism motion of the gun, and the changing velocity in a long displacement is measured by the PDV structure. The piston frame passes through the fixing device to connect the piston and the slider together to drive the actuator fixed on the slider to achieve reciprocating motion. In the experiment, a high-pressure gas pump was connected to one end of the air hole, and a two-position three-way valve was used to control the gas on-off. The test results show that the maximum speed of the sliding block can reach 12 m/s–14 m/s when one end of the sliding block is filled with air.

**Figure 5:**
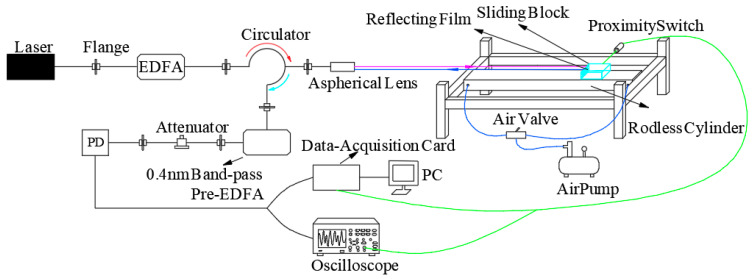
Block diagram of experimental system.

In the experiment, a rodless cylinder was used. The length of the guide rail was approximately 160 cm. A proximity switch was set near the air hole at one end. When the slider was pushed away from the proximity switch by high-pressure gas, an external trigger signal was generated. The effective movement length of the slider from the proximity switch to the other end of the cylinder can reach 147 cm. To improve the efficiency of laser reception, a reflective film is pasted on one side of the slider the structure of experimental system is as shown in Figure 5.

**Figure 6:**
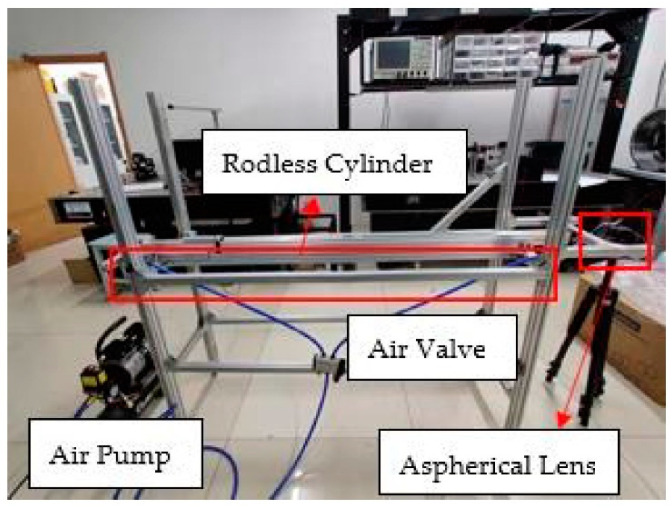
Arrangement of rodless-cylinder in experiment.

The slider of the air pump experiences four continuous processes: start-up, acceleration, deceleration and impact. When the slider hits the bottom of the guide rail, the speed does not return to zero immediately. The slider would vibrate for not only the support’s vibration but also the slider’s rebounding, leading to a phenomenon in which the speed would fluctuate after returning to zero and finally stop.The experiment site is as shown in Figure 6.

### 3.2. Analysis of Experimental Results

#### 3.2.1. Calibration Analysis of Single Point Frequency Data

Analysis of Data Correction Process:

The experiment uses a 200 MHz sampling rate data acquisition card to collect 25 million data points and uses the action of a proximity switch as the trigger. After collecting the data, the traditional MMFE is used to process the collected data. According to the results, the feasibility and operation method of correcting error frequency point selection are analyzed. Single point correction will be done based on analysis results.

**Figure 7:**
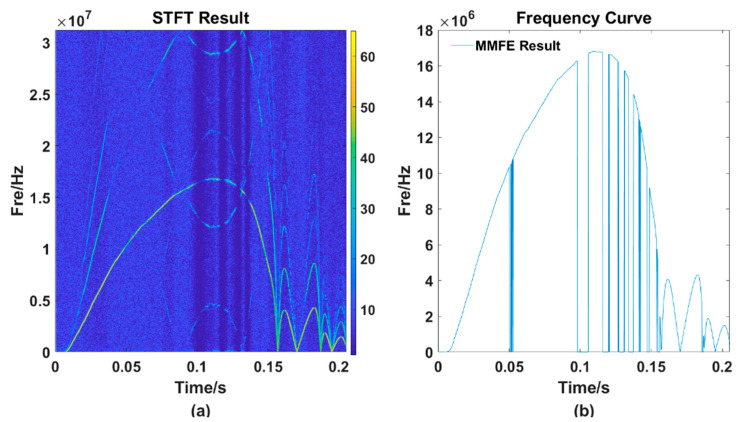
TF diagram processed by the traditional MMFE method. (**a**) the TF distribution obtained by the STFT method; (**b**) the TF curve obtained by the MMFE method.

**Figure 8:**
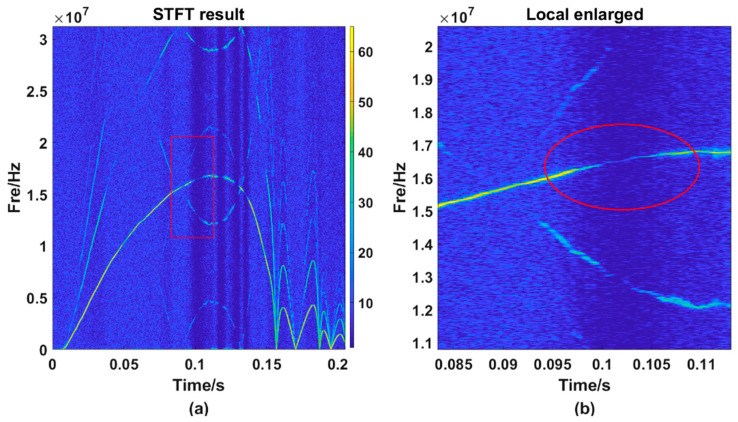
The local amplification diagram of one frequency selection error point. (**a**) the TF curve processed by the STFT method; (**b**) the partial enlargement of figure (**a**).

**Figure 9:**
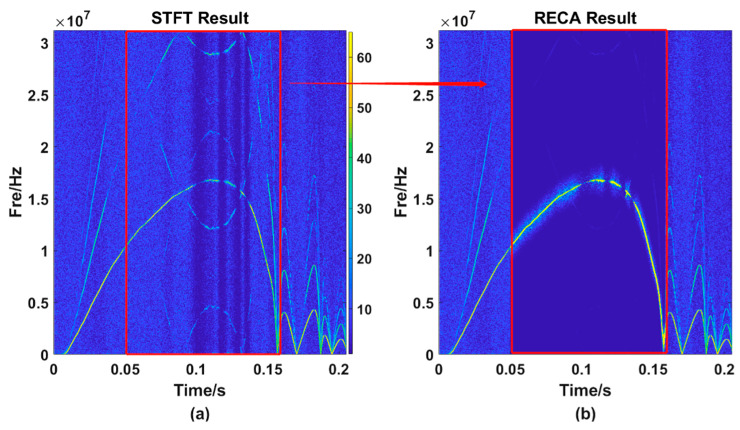
TF diagram obtained by using RECA. (**a**) the STFT result; (**b**) the RECA result.

The figures above shows that the TF distribution obtained by the STFT (Figure 7a) still have many energy components of other frequencies that interfere with the frequency selection, making the TF curve obtained by the MMFE (Figure 7b) have several jumping points. Magnify one of the incorrect frequency selection regions and we can get Figure 8:

It can be seen from Figure 8a that the main energy trajectory weakens at around 0.1 s, and the energy of other frequency points at the same time may exceed the value of the main energy trajectory. At this time, if the frequency is extracted according to the MMFE, the error will occur. Figure 8a is locally enlarged to obtain Figure 8b. It can be seen from the figure that, owing to the changes in light intensity and other interferences, the main energy trajectory becomes relatively weak. Necessary methods should be taken to strengthen the wrong point’s energy, in other words, to make the energy value of this wrong point stronger than other frequencies’ at the same time. We can obtain Figure 9b, the result after using RECA.

**Figure 10:**
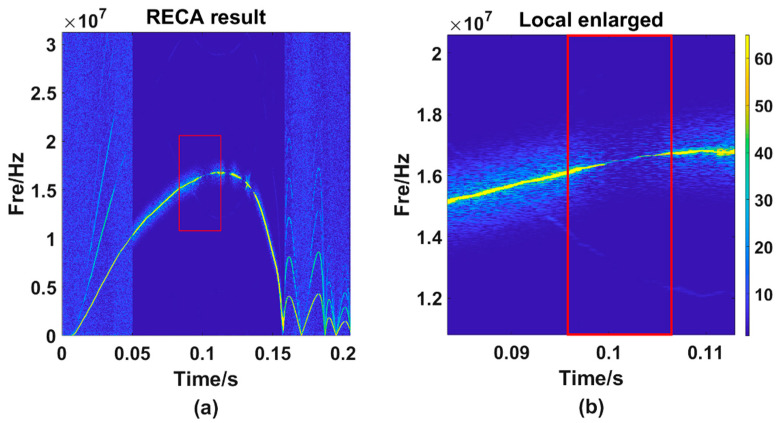
The local amplification diagram after correction of one frequency selection error point. (**b**) is the partial enlargement of RECA result (**a**).

In Figure 9a, data between 0.05 s and 0.16 s are corrected and then we could obtain Figure 9b. It is obvious that there is little energy remaining except within the main trajectory. Amplifying and observing the same region (as is shown in Figure 10a) selected before, we found that although the enhanced energy is still weaker than other points on the main trajectory, at the same time, the enhanced energy value is the strongest (as is shown in Figure 10b.).

**Figure 11:**
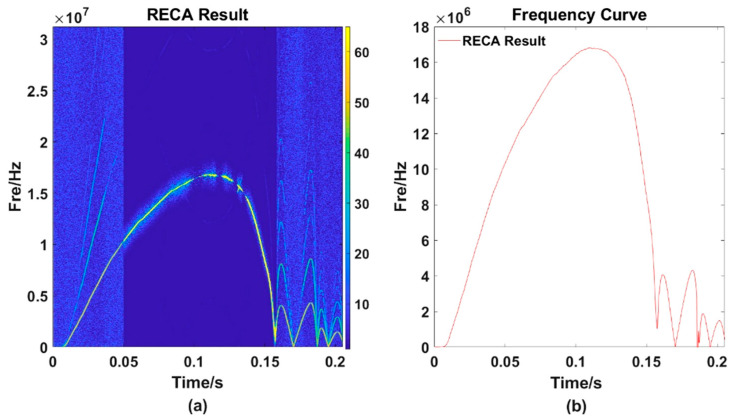
TF curve obtained by RECA. (**a**) the TF distribution result processed by RECA; (**b**) the TF curve.

Extract frequency points through the MMFE and then obtain a TF curve as is shown in Figure 11:

Examples of Single Point Data Correction Calculation Method:

Firstly, appropriate STFT parameters should be determined according to the characteristics of the experiment. The rodless cylinder is driven by compressed gas of 0.7 MPa pressure. Due to the damping factor of the cylinder itself, the speed curve of the slider is peak-like. The highest point of the speed appears in the middle part of the movement process, and the maximum acceleration part appears before the slider hits the end of the cylinder. Taking the TF curve in Figure 11 as an example, the motion time is about 0.15 s, and the maximum speed is about 14 m/s. Taking 1/5000 of the maximum speed as the speed resolution *f_res_*, which is 0.0028 m/s, and the corresponding frequency resolution could be calculated as 3.613 kHz. According to Equation (4), we could conclude that *N* ≥ 55,356. Since the premise of STFT is that the signal in the window is considered approximately stable, *N* should be raised to a power of 2 and as close to 55,536 as possible, for the closer the *N* is to 55,356, the higher the speed resolution is. The *t_res_* is determined by the overlap length of the sliding window. To ensure the *t_res_* of the time-speed curve and take into account the processing speed of the software, the overlap length of 55,536 is used for calculation in this example; that is, the time resolution is 50 μs. Due to the concentrated energy of laser interference Doppler signal, the selection of window function type should take into account the processing efficiency and ensure the low-frequency leakage phenomenon. In this case, the Hamming window with a small side lobe component is selected.

**Figure 12:**
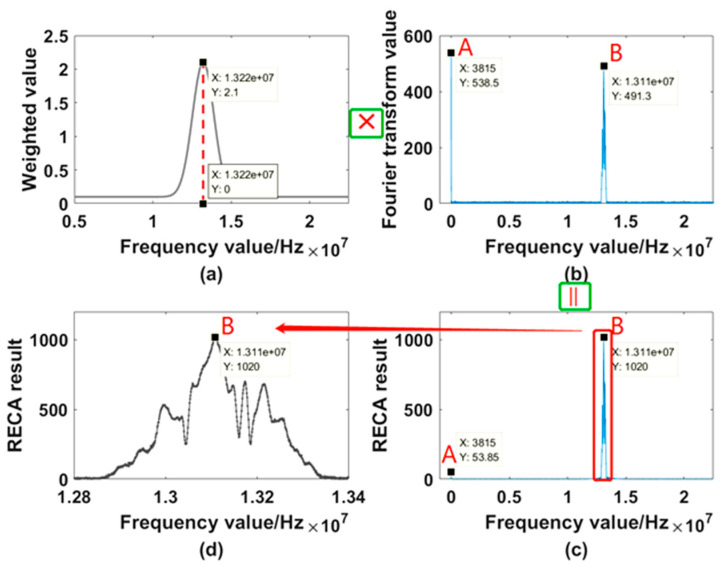
The correction calculation process of one error point. Points A and B are selected as an example to observe how the RECA works. (**a**) the weighted value distribution; (**b**) spectrum obtained by FFT; (**c**) spectrum acquired after using RECA; (**d**) partial enlargement of (**c**).

Take a set of data obtained in the actual test as an example. The window length of the system is set to 65,536. The frequency obtained by the previous FFT is set to 13.22 MHz, namely, the speed is 10.246 m/s. Generate energy weighting values at each frequency point of the next FFT using point 13.22 MHz as the center point. In this case, the self-weighted value is set to 0.1, the weighted value (the peak value of the Gaussian function) is set to 2, and the weighted width is set to 720. Then the weighted coefficient distribution of the next FFT spectrum modulus at each frequency point is shown in Figure 12a:

After obtaining the weighted values distribution, do FFT on the next set of data to obtain the spectrum as shown in Figure 12b. Because the point A has the maximum energy within the whole spectrum, the Figure 12b shows that the point A should be selected if we adopt traditional MMFE algorithm, the frequency of which is 3815 Hz, meaning a velocity of 0.03 m/s. The spectrum acquired after using RECA is shown in Figure 12c. It can be seen from the comparison between Figure 12b,c that the energy of the interference frequency (points near 0) far from the center point of 13.22 MHz has become very weak, so the range of the selected frequency is concentrated near 14 MHz. The Figure 12c is amplified to Figure 12d, it can be seen that after the correction, according to the maximum extraction frequency, the point will be selected at 13.11 MHz, that is, the speed of which is 10.16 m/s. Comparing the last point’s velocity 10.246 m/s with the current 10.16 m/s, we can see that it is obviously a deceleration process.

#### 3.2.2. Detailed Analysis of a Single Experiment Results

**Figure 13:**
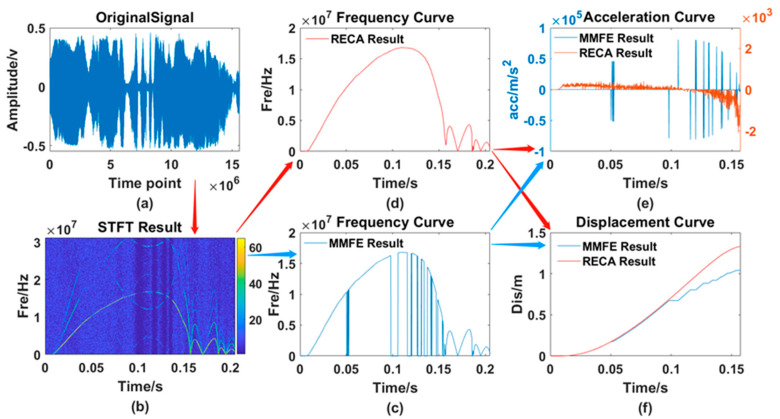
Comparison of results processed by two algorithms. (**a**) original signal; (**b**) TF distribution obtained by the STFT; (**c**) frequency curve processed by the MMFE; (**d**) frequency curve processed by the RECA; (**e**) acceleration curve; (**f**) displacement curve obtained by both the MMFE and RECA method.

After collecting the complete motion waveform data with the data acquisition card, the original waveform data is shown in Figure 13a; the whole TF diagram calculated by the traditional STFT is shown in Figure 13b. From Figure 13, the TF change curve obtained by the traditional MMFE, the velocity jump points can be clearly shown (i.e., the corresponding frequency jump, in other words, the frequency selection error). Due to the existence of such points, there is display error in the subsequent calculation of acceleration and accurate displacement, so it is necessary to use the RECA to recalculate the original data. The result calculated by the RECA is shown in Figure 13d. It can be seen from the comparison graph that the curve obtained by the new algorithm is smoother and more realistic than that obtained by the traditional algorithm.

In the process of the experiment, other relevant data obtained are also compared, such as displacement and acceleration, as shown in Figure 13e,f, which correspond to the displacement and acceleration of the test, respectively. The blue line in the figure represents the result obtained by using the MMFE, and the red line represents the result obtained by using the RECA. From the acceleration curve in Figure 13e, we can also see that the acceleration curve obtained by using the traditional MMFE has huge a fluctuation and jump at some micro points (as shown by the blue line), which is definitely wrong data for the speed of normal moving objects. After using the RECA, the acceleration curve becomes smooth and continuous. Therefore, the RECA can improve the stability of the system and the reliability of data selection compared to those of the traditional STFT algorithm.

#### 3.2.3. Comparative Analysis of Multiple Experimental Results

**Figure 14:**
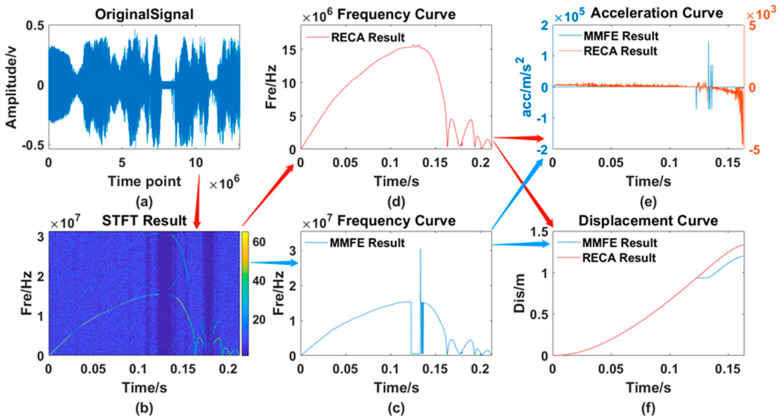
Comparison of the 1st set of experimental data. (**a**) original signal; (**b**) TF distribution obtained by the STFT; (**c**) frequency curve processed by the MMFE; (**d**) frequency curve processed by the RECA; (**e**) acceleration curve; (**f**) displacement curve obtained by both the MMFE and RECA method.

After testing the whole process of rodless cylinders many times, the two algorithms are used for processing. The results, including the original waveform, the processing result curve of the traditional MMFE and the processing result of the RECA are shown in Figure 14.

**Figure 15:**
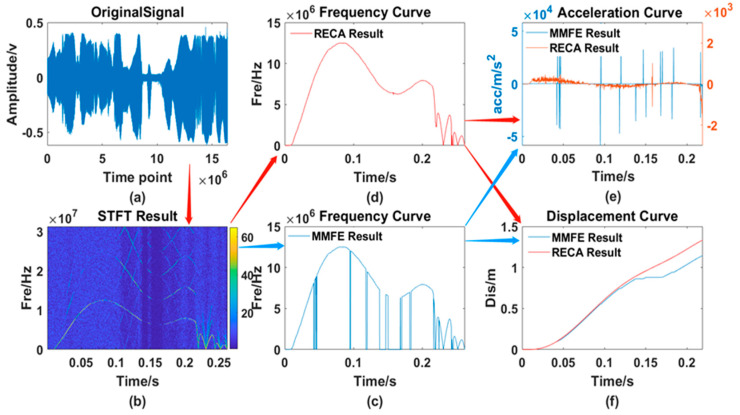
Comparison of the 2nd set of experimental data. (**a**) original signal; (**b**) TF distribution obtained by the STFT; (**c**) frequency curve processed by the MMFE; (**d**) frequency curve processed by the RECA; (**e**) acceleration curve; (**f**) displacement curve obtained by both the MMFE and RECA method.

**Figure 16:**
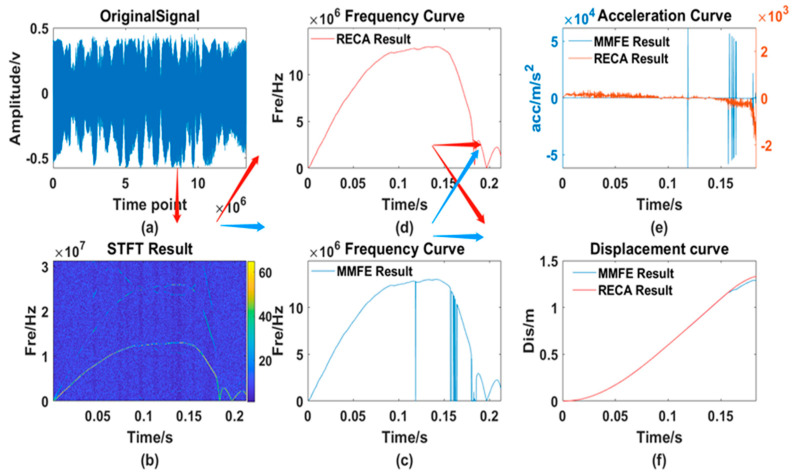
Comparison of the 3rd set of experimental data. (**a**) original signal; (**b**) TF distribution obtained by the STFT; (**c**) frequency curve processed by the MMFE; (**d**) frequency curve processed by the RECA; (**e**) acceleration curve; (**f**) displacement curve obtained by both the MMFE and RECA method.

From Figure 13, Figure 14, Figure 15 and Figure 16, 6 different graphs compose each Figure: Graph (a) represents the original waveform data; Graph (b) represents the TF distribution processed by STFT; Graph (c) represents the TF curve processed by traditional MMFE; Graph (d) represents the TF curve obtained by using RECA to correct the region with frequency selection error; Graph (e) and graph (f) represent the comparison between time-acceleration curve and time-displacement curve obtained by two algorithms, respectively, the blue lines of which show the data processed by the traditional MMFE, while the red lines show the data processed by the RECA.

According to our actual measurement results, the whole movement distance of the cylinder is 1360 mm. The difference between the distances calculated by the traditional MMFE and the actual distances range from 49 mm to 330 mm, while the difference between the results calculated by the RECA and the actual results is less than 8 mm, as shown in Table 1. The uncertainty Δ acts as

(9) $$\{\begin{array}{c} \Delta_{A}=\sqrt{\frac{\sum_{i=1}^{4}{\left(S_{i}-S\right)}^{2}}{3}}\\ \Delta_{B}=0.05\left(\mathrm{cm}\right)\\ \Delta =\sqrt{\Delta_{A}^{2}+\Delta_{B}^{2}} \end{array}$$

where uncertainty type *A* is evaluated by statistical methods, uncertainty type *B* is given by calibration reports. Value *S* is regarded as the true value of the length of the whole movement, namely 1360 mm. *S_i_* represents the value obtained by MMFE or RECA in No. (i) measurement.

According to the results in the above table, it is easy to find that the results calculated by the RECA method have less uncertainty than those calculated by the MMFE method, with a decrease of 97.07%. Therefore, this algorithm can play an important role in long-distance dynamic velocity measurement.

## 4. Conclusions and Prospect

The system has a stronger anti-disturbance ability due to the heterodyne method and the same optical path shared by both the signal and reference light. However, in the whole process of long displacement changing velocity measurement, owing to the influence of the fundamental wave, noise and light intensity jump, the results processed by the traditional MMFE still have the phenomenon of frequency jump (frequency selection error) at several time points. When only analyzing the trend of continuous variation of velocity, the influence is negligible, but the influence is nonnegligible when putting forward a high demand of velocity accuracy as there are velocity jump points, especially in measuring acceleration variation and accurate displacement. In order to reconstruct velocity curve with high dynamic range in long stroke, the problems should be solved when extracting TF ridge line. The RECA proposed in this paper can solve the problems of frequency selection error in the process of continuously measuring jumping velocity in a long displacement, such as the movement of gun automatic mechanism, or severe frequency selection jump in the process of nonstationary signal measurement, generating velocity curve more smoothly. As a result, the continuity of the velocity is much better, and the accuracy of displacement and acceleration is higher. These method’s advantages have been verified by numbers of experiments, having great significance in measuring velocity with long stroke and high dynamic range.

## Figures and Tables

**Table 1:** Comparison of displacement data in the motion process.

Serial Number	Measured Value	MMFEM easurements	Error Value (%)	Δ_MMFE_	RECA Measurements	Error Value (%)	Δ_RECA_	Error Reduction (%)
1	1360 mm	1212 mm	10.88	248.46	1354 mm	0.44	7.27	10.44
2	1360 mm	1030 mm	24.26		1353 mm	0.51		23.75
3	1360 mm	1132 mm	16.76		1352 mm	0.58		16.18
4	1360 mm	1311 mm	3.6		1357 mm	0.22		3.38

## Data Availability

Not applicable.
